# Chloroplast genome sequence of the wild *Ziziphus jujuba* Mill. var*. spinosa* from North China

**DOI:** 10.1080/23802359.2021.1878962

**Published:** 2021-03-01

**Authors:** Yuping Zhang, Guanglong Hu, Weitao Mao, Ningguang Dong, Bo Chen, Qinghua Pan

**Affiliations:** a Beijing Academy of Forestry and Pomology Sciences, Beijing, China; b Hubei Key Laboratory of Quality Control of Characteristic Fruits and Vegetables, College of Life Science and Technology, Hubei Engineering University, Xiaogan City, China

**Keywords:** *Ziziphus jujuba* Mill. var. spinosa, chloroplast genome, Illumina sequencing, phylogenetic analysis

## Abstract

In this study, the complete chloroplast (cp) genome sequence of *Ziziphus jujuba* Mill. var*. spinosa* was mapped and determined based on Illumina sequencing data. The complete cp genome is 161,606 bp and contains a pair of inverted repeat regions of 26,479 bp each, a large single-copy region of 89,292 bp, and a small single-copy region of 19,356 bp. It harbors 112 unique genes, including 78 protein-coding genes, 4 ribosomal RNA genes, and 30 transfer RNA genes. Phylogenetic analysis based on cp genomes indicates that the cp genome of wild *Z. jujuba* Mill. var. *spinosa* is similar to that of cultivated *Z. jujuba* and closely related to that of *Z. incurva* of the family *Rhamnaceae.*

The *Ziziphus* species (family *Rhamnaceae*) are plants considered to have dual medicinal and food purposes, and are distributed mainly in warm and subtropical regions throughout the world (Guo et al. 2011). Among them, the seeds of *Ziziphus jujuba* Mill. var. *spinosa* (Bunge) Hu ex H. F. Chou have traditionally been used as an ethnomedicine in Asian countries for thousands of years (Sun et al. 2011; Yang et al. 2013). Several chloroplast (cp) DNA markers have previously been used for the diversity analysis of *Z. jujuba* (Huang et al. 2015), and the whole genome of *Z. jujuba* has been sequenced (Liu et al. 2014; Huang et al. 2016). Nevertheless, there exists little genomic information about *Z. jujuba* Mill. var. *spinosa* from north China. In the present study, the complete cp genome sequence of *Z. jujuba* Mill. var. *spinosa* from north China is reported based on Illumina paired-end sequencing data (GenBank accession number: MW160433).

Fresh leaves were collected from a single *Z. jujuba* Mill. var. *spinosa* tree growing in Qinglonghu town (116°5′E, 39°47′N), Fangshan District, Beijing, China. Voucher specimens (accession number: ENC850490) were deposited at the herbarium of the Beijing Academy of Forestry and Pomology Sciences. DNA extraction was performed according to a modified CTAB protocol (Li et al. 2013). High-throughput sequencing was carried out using a HiSeq X Ten PE150 System (Illumina, San Diego, CA, USA). The cp genome was assembled with the SPAdes 3.6.1 (Bankevich et al. 2012) and Sequencher 4.10 (https://www.genecodes.com/) software tools. Reference-guided assembly was then performed to reconstruct the cp genome with the BLAST program (Altschul et al. 1990) using closely related species as references. After filling the gaps with GapCloser (http://soap.genomics.org.cn/), a 161,606-bp cp genome was obtained for *Z. jujuba* Mill. var. *spinosa.* Annotation was performed using the Plann script (Huang and Cronk 2015).

The circular cp genome of *Z. jujuba* Mill. var. *spinosa* contains a pair of inverted repeat (IR) regions of 26,479 bp each, and large single-copy (LSC) and small single-copy (SSC) regions of 89,292 bp and 19,356 bp, respectively. The genome comprises 112 unique genes, including 78 protein-coding genes, 30 transfer RNA genes, and 4 ribosomal RNA genes (16S, 23S, 5S, 4.5S). Among the annotated genes, 17 genes were found to contain introns, including 15 with a single intron each and two with two introns each (*clpP* and *ycf3*).

To identify the phylogenetic position of *Z. jujuba* Mill. var. *spinosa*, a maximum likelihood analysis was performed based on 24 complete chloroplast genomes using the Randomized Axelerated Maximum Likelihood (RAxML) program (Stamatakis 2014). The cp genome of *Z. jujuba* Mill. var. *spinosa* was found to be similar to that of cultivated *Z. jujuba* and closely related to that of *Z. incurva* of the family *Rhamnaceae* (Figure 1). This complete cp genome can be used for subsequent population and cp genetic engineering studies, and especially to determine the phylogenetic position of *Z. jujuba* Mill. var. *spinosa* in *Ziziphus* Mill.

**Figure 1.**
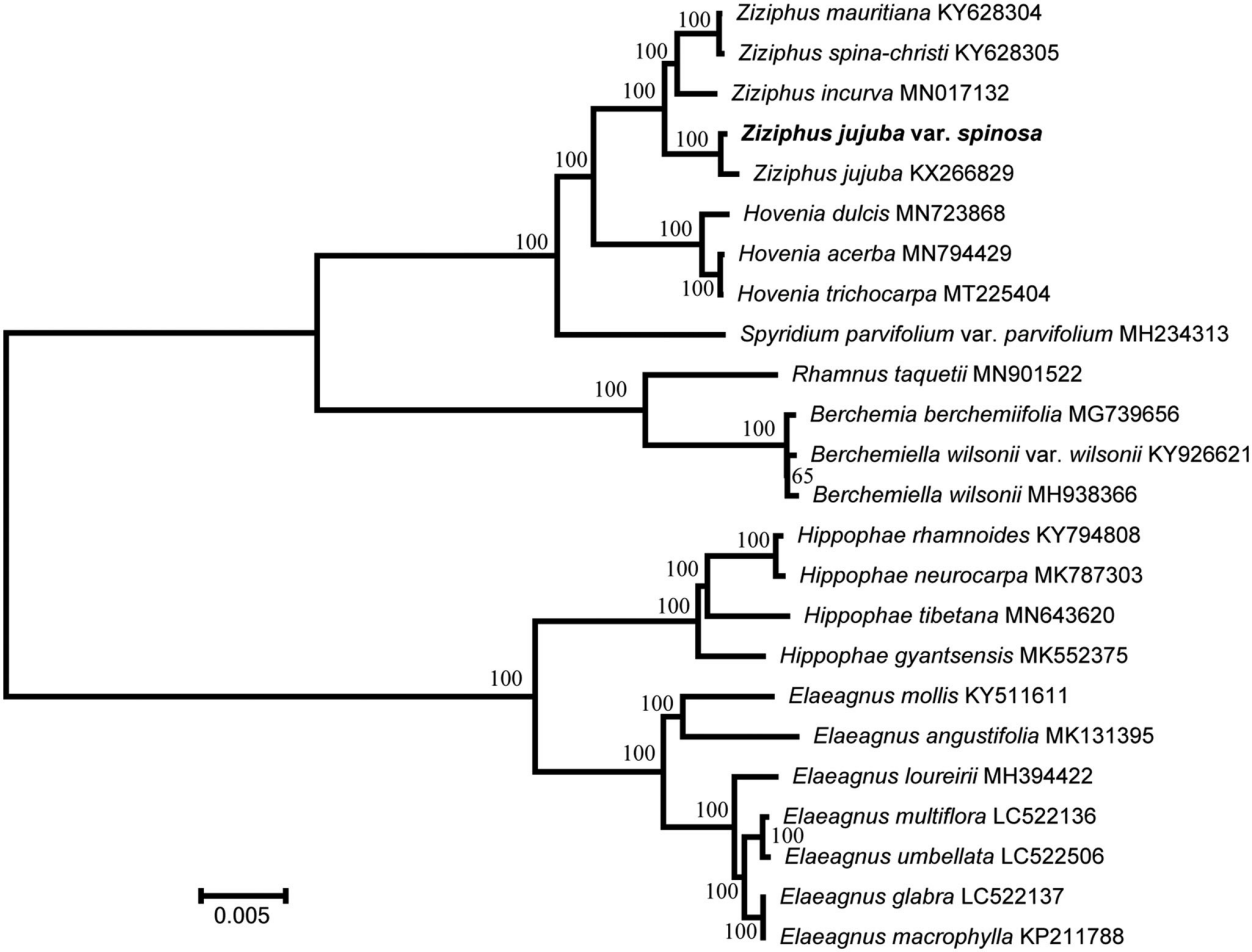
Phylogenetic tree inferred using the RAxML software from 24 complete chloroplast genomes.

## Data Availability

The genome sequence data that support the findings of this study are openly available in GenBank of NCBI at (https://www.ncbi.nlm.nih.gov/) under the accession no. MW160433.The associated BioProject, SRA, and Bio-Sample numbers are PRJNA684953, SRR13254568, SAMN17073062, respectively.
